# Exploring the key genes and pathways in enchondromas using a gene expression microarray

**DOI:** 10.18632/oncotarget.16700

**Published:** 2017-03-30

**Authors:** Zhongju Shi, Hengxing Zhou, Bin Pan, Lu Lu, Yi Kang, Lu Liu, Zhijian Wei, Shiqing Feng

**Affiliations:** ^1^ Department of Orthopaedics, Tianjin Medical University General Hospital, Tianjin, PR China

**Keywords:** enchondromas, microarray, differentially expressed genes, pathways, protein–protein interaction

## Abstract

Enchondromas are the most common primary benign osseous neoplasms that occur in the medullary bone; they can undergo malignant transformation into chondrosarcoma. However, enchondromas are always undetected in patients, and the molecular mechanism is unclear. To identify key genes and pathways associated with the occurrence and development of enchondromas, we downloaded the gene expression dataset GSE22855 and obtained the differentially expressed genes (DEGs) by analyzing high-throughput gene expression in enchondromas. In total, 635 genes were identified as DEGs. Of these, 225 genes (35.43%) were up-regulated, and the remaining 410 genes (64.57%) were down-regulated. We identified the predominant gene ontology (GO) categories and Kyoto Encyclopedia of Genes and Genomes (KEGG) pathways that were significantly over-represented in the enchondromas samples compared with the control samples. Subsequently the top 10 core genes were identified from the protein-protein interaction (PPI) network. The enrichment analyses of the genes mainly involved in two significant modules showed that the DEGs were principally related to ribosomes, protein digestion and absorption, ECM-receptor interaction, focal adhesion, amoebiasis and the PI3K-Akt signaling pathway.

Together, these data elucidate the molecular mechanisms underlying the occurrence and development of enchondromas and provide promising candidates for therapeutic intervention and prognostic evaluation. However, further experimental studies are needed to confirm these results.

## INTRODUCTION

Enchondromas are the most common primary benign osseous neoplasms of mature hyaline cartilage that occur within the medullary cavity of bone, and enchondromas account for 12 to 24 percent of all benign bone tumors and 3 to 10 percent of all bone tumors [1, 2]. Enchondromas occur predominantly in hands, typically in the middle and distal part of the metacarpals, and in the proximal part of the phalanges [3, 4]. Enchondromas can occur as solitary lesions or as multiple lesions (Ollier disease, Maffucci syndrome) [5, 6]. Multiple enchondromas can lead to malignant transformation more often (25-30%) than solitary enchondromas [7, 8]. The prevalence of Ollier's disease is estimated to be approximately 1 per 100, 000 people per year, and the incidence of malignant transformation has been estimated at 20-50% [9, 10]. Chondrosarcomas are the second most common type of malignant bone tumors after osteosarcomas [11]. Metastasis often takes place in high-grade chondrosarcomas and results in a poor survival rate [12]. Moreover, enchondromas and low-grade chondrosarcomas are usually histologically similar, so it is often impossible to distinguish them in clinics [13]. Therefore, finding out the molecular mechanism or biomarkers is necessary to help us understand the development of enchondromas and diagnose and treat enchondromas. Furthermore, targeting of key genes and pathways has been considered a promising approach in the diagnosis and treatment of cancer [14]. However, the molecular mechanism of enchondromas is unclear. Thus, identifying characteristic alerted genes and mechanisms associated with enchondromas is important for the inhibition of malignant transformation and the development of more effective therapies. Microarray techniques combined with bioinformatics analysis can determine the differential expression levels of genes accurately and provide an efficient method for large-scale gene expression studies [15]. Thus, analysis of gene expression profiling can provide a better understanding of molecular mechanisms and help to better diagnose or predict treatment response of patients with enchondroma.

In this study, by comparing the gene expression of enchondromas with normal growth plate and articular cartilage in the GEO database, we identified DEGs and performed Gene Ontology (GO) and Kyoto Encyclopedia of Genes and Genomes (KEGG) enrichment pathway analyses. In combination with protein–protein interaction (PPI) information, we not only identified relevant genes and pathways but also revealed existing molecular mechanisms. In conclusion, our analysis can improve our understanding of enchondroma and identify the key genes and pathways associated with diagnosis, prognosis, and treatment of enchondroma.

## RESULTS

### Identification of DEGs

**Figure 1:**
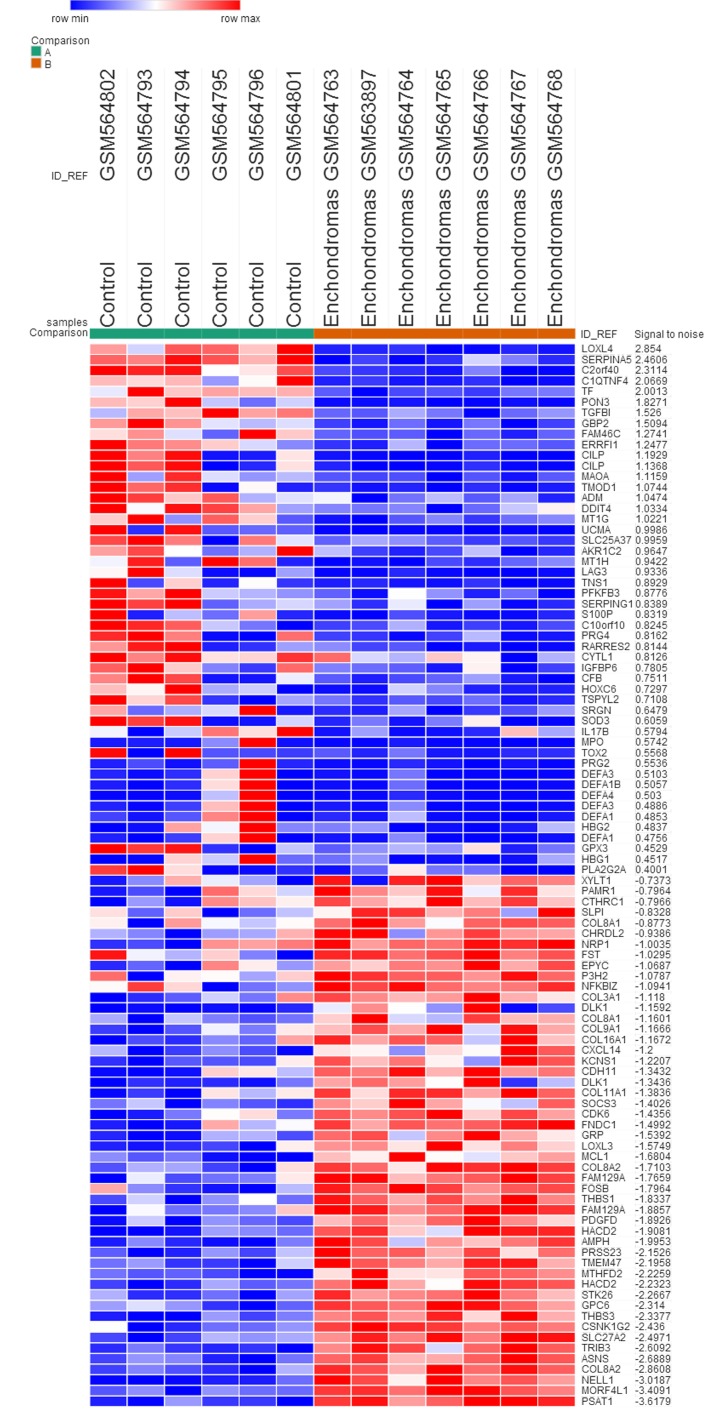
The heat map of the DEGs (top 50 up-regulated and down-regulated genes) Enchondromas samples versus control samples. Changes in genes expression (*P* < 0.05, logFC > 1.0 or logFC < -1.0) are illustrated by a heat map. Blue indicates a relatively low expression and red indicates a relatively high expression.

The gene expression profile GSE22855 was downloaded from the GEO database, and the GEO2R method was used to identify DEGs in enchondroma samples compared with control samples. We used *P* < 0.05, logFC (fold control) > 1.0 or logFC < -1.0 as the criteria, and 635 genes were identified as DEGs. Among these, 225 genes (35.43%) were up-regulated, and the remaining 410 genes (64.57%) were down-regulated. Subsequently, we created the heatmap using the top 50 up-regulated and down-regulated DEGs (Figure 1).

### GO term enrichment analysis

Then, the online software DAVID was used to functionally categorize these 635 significant DEGs. GO analysis revealed that the up-regulated DEGs were significantly enriched in BP, including defense response to fungus, response to fungus, and defense response to Gram-positive bacterium (Table 1); the down-regulated DEGs were significantly enriched in BP, including extracellular matrix organization, extracellular structure organization, and collagen metabolic process (Table 1). For MF, the up-regulated DEGs were enriched in sulfur compound binding, glycosaminoglycan binding and heparin binding, and the down-regulated DEGs were enriched in extracellular matrix structural constituent, structural molecule activity, and growth factor binding (Table 1). In addition, GO CC analysis also showed that the up-regulated DEGs were significantly enriched in the extracellular space, extracellular region part, and extracellular region, and down-regulated DEGs were enriched in the extracellular region part, extracellular region and extracellular matrix (Table 1).

**Table 1:** Gene ontology analysis of DEGs

Expression	Category	GO-ID	Term	Gene count	%	*P* value
Up-regulated	BP	GO:0050832	Defense response to fungus	11	5.6	9.20E-12
	BP	GO:0009620	Response to fungus	11	5.6	3.00E-10
	BP	GO:0050830	Defense response to Gram-positive bacterium	12	6.1	3.00E-09
	BP	GO:0042742	Defense response to bacterium	18	9.1	7.00E-09
	BP	GO:0009617	Response to bacterium	25	12.7	2.30E-08
	MF	GO:1901681	Sulfur compound binding	16	8.1	4.30E-08
	MF	GO:0005539	Glycosaminoglycan binding	15	7.6	6.20E-08
	MF	GO:0008201	Heparin binding	13	6.6	1.80E-07
	MF	GO:0050786	RAGE receptor binding	4	2	1.90E-04
	MF	GO:0016209	Antioxidant activity	7	3.6	1.90E-04
	CC	GO:0005615	Extracellular space	56	28.4	1.10E-15
	CC	GO:0044421	Extracellular region part	89	45.2	4.00E-12
	CC	GO:0005576	Extracellular region	96	48.7	9.00E-11
	CC	GO:0070062	Extracellular exosome	68	34.5	1.10E-09
	CC	GO:1903561	Extracellular vesicle	68	34.5	1.40E-09
Down-regulated	BP	GO:0030198	Extracellular matrix organization	37	11.6	3.00E-18
	BP	GO:0043062	Extracellular structure organization	37	11.6	3.30E-18
	BP	GO:0032963	Collagen metabolic process	20	6.3	1.30E-13
	BP	GO:0044259	Multicellular organismal macromolecule metabolic process	20	6.3	2.80E-13
	BP	GO:0044259	Multicellular organism metabolic process	20	6.3	3.90E-12
	MF	GO:0005201	Extracellular matrix structural constituent	16	5	1.20E-11
	MF	GO:0005198	Structural molecule activity	43	13.5	9.00E-11
	MF	GO:0019838	Growth factor binding	16	5	7.90E-09
	MF	GO:0030020	Extracellular matrix structural constituent conferring tensile strength	6	1.9	9.90E-09
	MF	GO:0005102	Receptor binding	49	15.4	2.00E-05
	CC	GO:0044421	Extracellular region part	159	50	1.10E-25
	CC	GO:0044421	Extracellular region	171	53.8	3.10E-23
	CC	GO:0031012	Extracellular matrix	53	16.7	3.40E-22
	CC	GO:0005578	Proteinaceous extracellular matrix	43	13.5	8.60E-21
	CC	GO:0005788	Endoplasmic reticulum lumen	29	9.1	2.50E-16

Abbreviations: BP, Biological process; MF, Molecular function; CC, Cellular component.

### KEGG pathway analysis

The result of KEGG analysis revealed that DEGs were enriched in ECM-receptor interaction, protein digestion and absorption, focal adhesion, PI3K-Akt signaling pathway and ribosomes, and the key genes involved in these pathways are summarized in Table 2.

**Table 2:** KEGG pathway analysis of DEGs

Pathway-ID	Name	Gene count	%	*P* value	Genes
4512	ECM-receptor interaction	18	3.5	3.60E-09	CHAD, COL1A1, COL1A2, COL2A1, COL3A1, COL4A1, COL4A2, COL6A3, COL11A1, COL11A2, ITGB5, LAMB2, TNC, THBS1, THBS2, THBS3, THBS4, VWF
4974	Protein digestion and absorption	16	3.1	2.10E-07	FXYD2, COL1A1, COL1A2, COL2A1, COL3A1, COL4A1, COL4A2, COL9A1, COL9A2, COL9A3, COL6A3, COL11A1, COL11A2, COL12A1, PRCP, SLC7A8
4510	Focal adhesion	23	4.5	1.60E-06	JUN, CAV1, CAV2, CHAD, COL1A1, COL1A2, COL2A1, COL3A1, COL4A1, COL4A2, COL6A3, COL11A1, COL11A2, CCND3, ITGB5, LAMB2, PDGFD, TNC, THBS1, THBS2, THBS3, THBS4, VWF
4151	PI3K-Akt signaling pathway	27	5.2	1.00E-04	MCL1, DDIT4, ATF4, CHAD, COL1A1, COL1A2, COL2A1, COL3A1, COL4A1, COL4A2, COL6A3, COL11A1, COL11A2, CCND3, CDK6, ITGB5, IFNA8, LAMB2, PCK2, PDGFD, RPS6, TNC, THBS1, THBS2, THBS3, THBS4, VWF
3010	Ribosome	15	2.9	2.00E-04	RPL13, RPL14, RPL21, RPL23, RPL37A, RPL5, RPL7, RPL7A, RPS15, RPS15A, RPS2, RPS28, RPS3A, RPS6, RPLP1

### PPI Network of DEGs and core genes in the PPI network

**Figure 2:**
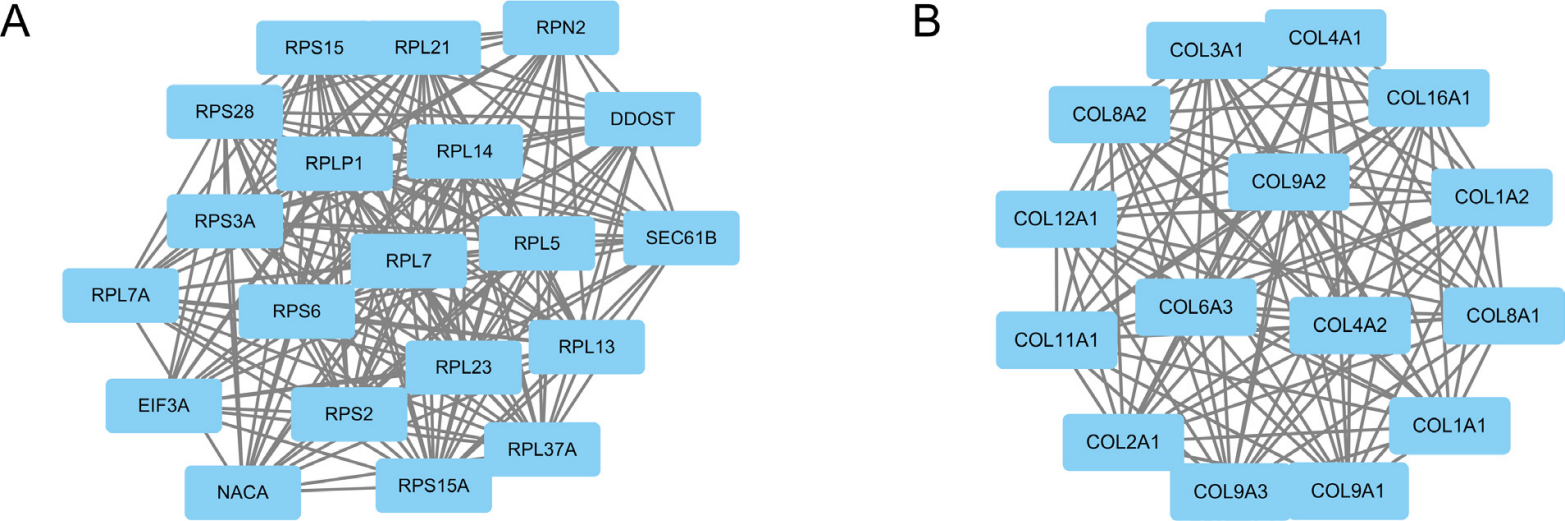
Top two modules from the PPI network The squares represent the DEGs in modules, and the lines show the interaction between the DEGs. The top two modules indicate that they may play a more important role in the PPI network. **(A)** module 1. **(B)** module 2.

Based on the information in the STRING database, the PPI network contained 393 nodes and 1534 edges. The nodes indicated the DEGs, and the edges indicated the interactions between the DEGs. The top 10 high-degree hub nodes included glyceraldehyde-3-phosphate dehydrogenase (GAPDH), Jun proto-oncogene (JUN), matrix metallopeptidase 9 (MMP9), Fos proto-oncogene (FOS), collagen type I alpha 1 chain (COL1A1), early growth response 1 (EGR1), collagen type II alpha 1 chain (COL2A1), thrombospondin 1 (THBS1), forkhead box O1 (FOXO1) and collagen type I alpha 2 chain (COL1A2). Among these genes, GAPDH showed the highest node degree, which was 87. The core genes and their corresponding degree are shown in Table 3. Then, we used MCODE to screen the modules of the PPI network (Figure 2), and we performed an enrichment analysis of the genes involved in the top two significant modules. The results showed that the DEGs in modules 1 and 2 were principally related to ribosomes, protein digestion and absorption, ECM-receptor interaction, focal adhesion, amoebiasis and PI3K-Akt signaling pathway (Table 4).

**Table 3:** The core genes and their corresponding degree

Gene	Degree	Gene	Degree	Gene	Degree	Gene	Degree
GAPDH	87	EGR1	35	RUNX2	31	RPS2	26
JUN	61	COL2A1	35	CTGF	29	RPL5	26
MMP9	56	THBS1	32	RPS6	28	RPS15	26
FOS	52	FOXO1	32	COL3A1	28	HSPA8	26
COL1A1	41	COL1A2	31	VWF	27	COL4A1	26

**Table 4:** The enriched pathways of modules

Modules	Enriched pathways	*P* value	False discovery rate	Nodes
1	Ribosome	1.90E-21	1.64E-27	RPL13, RPL14, RPL21, RPL23, RPL37A, RPL5, RPL7, RPL7A, RPS15, RPS15A, RPS2, RPS28, RPS3A, RPS6, RPLP1
2	Protein digestion and absorption	7.50E-22	1.31E-24	COL1A1, COL1A2, COL2A1, COL3A1, COL4A1, COL4A2, COL9A1, COL9A2, COL9A3, COL6A3, COL11A1, COL12A1
	ECM-receptor interaction	1.20E-11	5.71E-14	COL1A1, COL1A2, COL2A1, COL3A1, COL4A1, COL4A2, COL6A3, COL11A1
	Focal adhesion	5.60E-09	3.42E-11	COL1A1, COL1A2, COL2A1, COL3A1, COL4A1, COL4A2, COL6A3, COL11A1
	Amoebiasis	4.90E-09	3.42E-11	COL1A1, COL1A2, COL2A1, COL3A1, COL4A1, COL4A2, COL11A1
	PI3K-Akt signaling pathway	3.20E-07	1.63E-09	COL1A1, COL1A2, COL2A1, COL3A1, COL4A1, COL4A2, COL6A3, COL11A1

## DISCUSSION

Enchondromas are benign cartilaginous neoplasms that commonly occur in medullary bone [1]. These tumors can occur at any age, and they are found most commonly in children and adolescents with no significant sex differences [16]. Additionally enchondromas can undergo malignant transformation into chondrosarcoma [17, 18]. However, because they are clinically silent, enchondromas remain undetected in patients, and few studies address the molecular mechanism of enchondromas [19]. In the current study, the gene expression profile of GSE22855 was downloaded and a bioinformatic analysis was performed. The analysis results showed that there were 635 differentially expressed genes in the enchondroma samples compared with the control samples. Furthermore, GO, KEGG pathway and PPI analyses were performed to obtain a better understanding of this tumor, which was easily ignored.

The results of GO analyses showed that the significant ontology categories of up-regulated DEGs included defense response to fungus, sulfur compound binding and heparin binding. A previous study showed that garlic can detoxify carcinogens by stimulation of sulfur compound binding [20]. Moreover, up-regulation of genes associated with glycosaminoglycan binding was also found in ovarian cancer [21]. A recent study suggested that blocking heparan sulfate (HS) interaction with the heparin-binding domains of fibroblast growth factor receptors (FGFRs) could inhibit cancer cell growth [22]. Furthermore, down-regulated DEGs were mainly related to extracellular matrix organization, the extracellular matrix structural constituent and the collagen metabolic process. Elevation of genes associated with extracellular matrix organization could also be found in well-differentiated squamous cell carcinoma and colorectal cancer [23, 24]. Another analysis of secretome profiles on cancer-associated fibroblasts showed that up-regulated proteins were involved mainly in extracellular matrix organization and disassembly and collagen metabolism [25]. Therefore, GO analysis can help identify the possible biological processes, molecular functions and cellular components involved in the occurrence and development of enchondromas.

Moreover, the KEGG pathways revealed that DEGs were enriched in ECM-receptor interaction, protein digestion and absorption, focal adhesion, the PI3K-Akt signaling pathway and ribosomes. As a member of the basic helix-loop-helix (bHLH) family which plays a key role in tumorigenesis, Twist2 could promote proliferation and invasion of kidney cancer cell via regulating the ECM-Receptor-Interaction pathway [26]. The protein digestion and absorption pathway has been reported to be associated with pancreatic neuroendocrine tumors and breast cancer [27, 28]. Focal adhesion has been verified as taking part in cell migration in various tumor cells [29–31]. The phosphatidylinositol-3 kinases/Akt (PI3K/Akt) signaling pathway is activated in many human tumors, and it was proved to be a promising anticancer target [32]. Thus, deep understanding of the pathways can help us to elucidate the crucial mechanism of enchondromas.

Furthermore, we analyzed the PPI network and found that GAPDH, JUN, MMP9, FOS, COL1A1, EGR1, COL2A1, THBS1, FOXO1 and COL1A2 were the top 10 core genes, which may be potential therapeutic targets for enchondromas. GAPDH showed the highest node degree among these genes. GAPDH is deregulated in various cancer cells, and it is a new therapeutic target associated with tumor progression [33]. Therefore, experimental studies on GAPDH are essential to understand its role in the molecular mechanisms of enchondromas. C-jun was an important oncogene that could provide signals for cell survival; it was highly overexpressed in various invasive cancers, and repression of c-jun was beneficial to inhibiting the development and progression of cancer [34, 35]. MMP9 has been shown to be involved in the migration and invasion of various tumors, including breast cancer, transitional cell carcinoma, and non-small cell lung cancer [36–39]. A recent study revealed that epigenetic down-regulation of COL1A1 mRNA expression might have a role as a prognostic biomarker of hepatocellular carcinoma [40]. Expression profiles of COL2A1 and COL1A2 were independent predictors of survival in ovarian cancer and head and neck cancer respectively [41, 42]. EGR1 isn't only a tumor suppressor gene (TSG) but also a gene with oncogenic activities [43], so understanding how it changes in enchondromas helps us to elucidate the crucial mechanism of tumorigenesis. As a tumor-specific ECM protein, THBS1 could promote migration of cancer cells and cause activation of integrin signaling in oral squamous cell carcinoma [44]. FOXO1 was a key effector of PI3K/Akt signaling and functions as tumor suppressors [45]. Therefore, the analysis of these core genes is useful for understanding the molecular mechanisms and identifying therapeutic targets of enchondromas.

Moreover, two main modules were got from the module analysis of the PPI network, and enrichment analysis showed that the modules were involved in ribosomes, protein digestion and absorption, ECM-receptor interaction, focal adhesion, amoebiasis and the PI3K-Akt signaling pathway. These results agreed with those of the KEGG analysis, and these related pathways represented promising candidates for therapeutic intervention and prognostic evaluation.

Somatic mutations in isocitrate dehydrogenase 1 (IDH1) and isocitrate dehydrogenase 2 (IDH2) have been detected in secondary glioblastomas, gliomas and acute myeloid leukemia (AML) [46–48]. IDH1 and IDH2 mutations are identified as the first common genetic abnormalities in conventional central and periosteal cartilaginous tumors, including enchondromas [49]. IDH1 mutations usually result in substitutions at R132, whereas IDH2 mutations affect R172, R132 and R140 [49, 50]. Moreover, the mutations occur in the early stages of tumorigenesis and can cause the accumulation of D-2-hydroxglutarateare [51]. The overall frequency of IDH1/IDH2 mutations was approximately 56% in conventional central and periosteal cartilaginous neoplasms [49]. Furthermore, a previous study showed that DNA hypermethylation was a consequence of IDH1/IDH2 mutations in AML and could result in reduced haemopoietic cellular differentiation and loss of markers related to proliferation [52]. Previous studies have demonstrated both positive and negative correlations between intragenic DNA methylation and gene expression, and abnormal DNA methylation was considered a common mechanism in the pathogenesis of several types of tumors [53–56]. Therefore, the researches on the impact of DNA methylation on gene expression in enchondromas are quite necessary. In our analysis, IDH1 and IDH2 were also identified as DEGs, and the results of these previous studies provided support for the result of our analysis. Furthermore, it is essential to explore the significant mutations and aberrant DNA methylation that occur in enchondromas, as they appear to be helpful for understanding the genetic alterations and molecular mechanisms of enchondromas.

SNP arrays are a useful research tool in molecular biology that can provide an analysis of DNA copy number alterations (CNA) and loss of heterozygosity (LOH); it can also detect genetic alterations in tumors [57, 58]. A previous study used the SNP array in combination with an expression array and aimed to obtain a comprehensive registry of genetic aberrations; the results demonstrated that CNA and LOH are rare and non-recurrent in enchondromas [59]. This study mainly studied genetic alterations including CAN and LOH, while our study mainly studied the global changes in gene expression and attempted to explore the molecular mechanisms underlying enchondromas. Furthermore, another study performed immunohistochemical analysis and quantitative real-time polymerase chain reaction (PCR) to reach a better understanding of the molecular mechanisms underlying malignant transformation of enchondromas; the result demonstrated that parathyroid hormone related peptide (PTHrP) signaling is active in enchondromas, and the PTH type 1 receptor (PTHR1) and B-cell lymphoma-2 (Bcl-2) were associated with tumor progression [7]. Another article performed a genome-wide cDNA expression analysis and found that Ollier's disease and solitary enchondromas revealed similar expression profiles; JunB may be of diagnostic relevance to grade I chondrosarcomas [60]. This result was also supported by our study that JunB was also a DEG in our study. In addition, the increase in glycolysis-associated genes and decrease in oxidative phosphorylation-related genes was found in high-grade chondrosarcoma and these genes were considered to be associated with chondrosarcoma progression [60]. Compared with previous studies, the present study found several novel genes, such as GAPDH, MMP9, FOS, COL1A1 and EGR1, which might be potentially associated with enchondromas. We also discovered potential PPIs between these genes, and if the roles of these genes in enchondromas are confirmed, the genes could potentially be utilized in the molecular diagnosis or treatment selection of enchondromas.

This study had several limitations. First, the sample sizes for the expression profiling were not large, so further studies with larger sample sizes are needed to verify the results. Second, it is acknowledged that predicting key genes merely by means of bioinformatics is not sufficient, and further molecular biological experiments are needed to confirm these results. Therefore, we hope that these data can be incorporated into future experiments; the results can give us a better understanding of the molecular mechanisms and provide novel biomarkers for the molecular therapy of enchondromas. Despite these limitations, we believe that this analysis represents a valuable resource and may be meaningful for further diagnosis and therapy for this disease.

## CONCLUSION

In conclusion, the present study identified 635 DEGs, which may be involved in the occurrence and development of enchondromas via comprehensive bioinformatics analysis. GO term, KEGG pathway and PPI network analyses provided a set of related genes and pathways to help elucidate the molecular mechanisms of enchondromas. Further experimental studies are needed to confirm these results and should help determine potential targets for diagnosis, prognosis, and treatment of enchondroma.

## MATERIALS AND METHODS

### Gene expression microarray data

In current study, the gene expression profiles of GSE22855 were downloaded from Gene Expression Omnibus (GEO, http://www.ncbi.nlm.nih.gov/geo/). GSE22855 was based on Illumina Inc GPL6884 platform (Illumina HumanWG-6 v3.0 expression beadchip). The GSE22855 dataset contained 13 samples, including 7 enchondromas samples and 6 control samples.

### Identification of DEGs

The raw data files used for the analysis included TXT files (Illumina platform). The analysis was carried out using GEO2R, which can perform comparisons on original submitter-supplied processed data tables using the GEOquery and limma R packages from the Bioconductor project. The DEGs between the enchondromas samples and control samples were selected (P value < 0.05), and overlapped genes with statistical significance were identified.

### GO enrichment and KEGG pathway analysis of the DEGs

After identifying the DEGs, we submitted the DEGs list to the online software Database for Annotation, Visualization and Integrated Discovery (DAVID, https://david.ncifcrf.gov/) to identify overrepresented GO categories and pathway categories. GO analysis can provide quantitative and statistical output files to determine the biological meaning in a large list of genes and categorize gene product functions, including biological process (BP), molecular function (MF) and cellular component (CC) [61, 62]. KEGG (http://www.genome.jp/) is a knowledge base for systematic analysis of gene functions, linking genomic information with higher-level systemic functions [63, 64]. Finally, the enriched functions of DEGs were selected via GO and KEGG pathway analysis, and *P* < 0.05 was considered statistically significant.

### Construction of the PPI network of DEGs

To further investigate the molecular mechanism of enchondromas in development and progression, we used the Search Tool for the Retrieval of Interacting Genes (STRING) database (http://www.string-db.org/) to evaluate the interactive relationships among DEGs. We first submitted the DEGs list to STRING, and then, we selected the experimentally validated interactions with a combined score > 0.4. Subsequently, the PPI networks were analyzed using Cytoscape software. Then, the plug-in Molecular Complex Detection (MCODE) was applied to screen the modules of the PPI network in Cytoscape. Furthermore, the enrichment analyses were performed for DEGs in the corresponding modules. *P* < 0.05 was considered statistically significant.
